# Association of hemoglobin-to-red cell distribution width ratio with the three-month outcomes in patients with acute ischemic stroke

**DOI:** 10.3389/fneur.2024.1425633

**Published:** 2024-08-12

**Authors:** Xiaorui Xie, Keli He, Yao Zhang, Jianhua Wu

**Affiliations:** ^1^Department of Neurology, Xiangya Changde Hospital, Changde, Hunan, China; ^2^Department of Clinical Laboratory, Changde Hospital, Xiangya School of Medicine, Central South University (The First People’s Hospital of Changde City), Changde, Hunan, China

**Keywords:** stroke, three-month outcomes, hemoglobin-to-red cell distribution width ratio, nonlinearity, prognosis

## Abstract

**Aim:**

To explore the association of Hemoglobin-to-Red Cell Distribution Width Ratio (HRR) with the risk of three-month unfavorable outcomes in acute ischemic stroke (AIS).

**Methods:**

A secondary analysis was conducted based on a prospective cohort study. A total of 1,889 patients with AIS treated in South Korea from January 2010 to December 2016 were enrolled. Multivariable logistic regression was conducted to investigated the independent relationship between HRR and risk of three-month unfavorable outcomes in AIS. Fitted smoothing curves were used to determine non-linear correlations. The recursive method was employed to explore the turning point and build a two-piece linear regression model. In addition, a set of subgroup analyses were carried out to evaluate the relationship between HRR and risk of three-month unfavorable outcomes.

**Results:**

Multivariate analysis in which potential confounders were adjusted for indicated that the risk of unfavorable outcomes was reduced by 10% for each unit increased of HRR [OR = 0.90, 95% CI: 0.84–0.96, *p* = 0.0024]. In addition, a non-linear relationship was observed between HRR and risk of three-month unfavorable outcomes, which had an inflection point of HRR was 10.57. The effect sizes and the confidence intervals on the left side of the inflection point were 0.83 (0.75, 0.91), *p* = 0.0001. On the right side of the inflection point, no association was found between HRR and the risk of three-month unfavorable outcomes.

**Conclusion:**

This study demonstrates a negative association between HRR and risk of three-month unfavorable outcomes. The relationship between HRR and risk of three-month unfavorable outcomes is non-linear. The correlation is negative for HRR values less than 10.57. For, HRR higher than 10.57, HRR is not associated with the risk of three-month unfavorable outcomes.

## Introduction

Stroke is the second leading cause of death worldwide, with acute ischemic stroke (AIS) accounting for approximately 80% of all stroke cases (1). Although it has high mortality and recurrence rates, AIS can be prevented. Early screening and implementation of effective interventions can slow down the progression of AIS and early neurological deterioration, thereby improve patient outcomes.

The ratio of hemoglobin to red blood cell distribution width (HRR) is a simple and robust biomarker of inflammation calculated from hemoglobin and red blood cell distribution width (RDW).

Several studies have demonstrated that HRR contribute to the occurrence, development and prognosis of various diseases (2–7). Qin et al. reported a relationship between HRR and mortality in AIS patients with atrial fibrillation (8). Eyiol and Ertekin have shown that a low HRR measured upon hospital admission is a valuable marker for predicting stroke mortality and determining stroke severity (9). For stroke outcome assessment, several guidelines recommend using the modified Rankin Scale (mRS) score at 3 months as the preferred endpoint due to its strengths in evaluating functional status (10). Accurate prediction of functional outcomes in stroke patients can improve treatment interventions, guide patient and family education, and optimize rehabilitation and discharge planning (11). However, to our knowledge, none study conducted statistical analysis to determine the association of HRR with the risk of three-month unfavorable outcomes in AIS patients. Hence, we conducted a secondary analysis of data from a cohort of 1906 study to generate evidence to guide future application of HRR in prognostic evaluation of AIS patients.

## Materials and methods

### Study population

This was a second analysis based on data from a prospective cohort study (12). The original study examined 2,084 patients with acute ischemic stroke who were admitted within 7 days of symptom onset using the single-center prospective registry method. In the study, participants who lacked laboratory information or dysphagia test within 24 h of admission, and the modified 3-month mRS score data after hospitalization were excluded. In our study, extreme outliers greater or less than 3-fold standard deviations from the mean were excluded.

### Variables

The general characteristics of the study cohort including age, sex, body mass index (BMI), white blood cells (WBC), platelet (PLT), total serum cholesterol (TC), serum triglyceride (TG), serum high-density lipoprotein cholesterol (HDL-c), serum low-density lipoproteins cholesterol (LDL-c), serum creatinine (Scr), Blood urea nitrogen (BUN), aspartate aminotransferase (AST), alanine aminotransferase (ALT), hemoglobin concentration (HGB), Red cell distribution width (RDW), diabetes mellitus (DM), Hyperlipidemia, atrial fibrillation (AF), Coronary heart disease(CHD), hypertension, Smoking, and National Institutes of Health Stroke Scale (NIHSS) scores were recorded.

We selected the HRR as the independent variable while the dependent variable was the 3-month function outcome in patients with AIS (dichotomous variable: unfavorable outcome, favorable outcome).

Three-month outcomes after AIS onset were determined based on the mRS score. Participants were divided into two groups: favorable outcomes and unfavorable outcomes. The unfavorable outcomes was defined as mRS score ≥ 3; the favorable outcomes was defined as the mRS score < 3.

### Statistical analyses

For continuous variables, we reported the mean values plus the standard deviations (SD) to show variability. To identify any significant differences in three-month outcomes among AIS patients, we employed statistical tests appropriate for the data distribution: one-way ANOVA for normally distributed data, Kruskal-Wallis H test for skewed data, and the chi-square test for categorical variables. Univariate and multivariate logistic regression models were utilized to estimate the correlation between HRR and the risk of three-month outcomes. Besides, we analyzed the effect of confounding factors on the model and provided three adjusted models. The results are presented as hazard ratio (HR) with 95% confidence intervals (CI). In model I, no covariates were adjusted. In model II, only age and sex were adjusted. In model III, all possible confounders were adjusted. We assessed potential confounding factors by looking for changes in the effect estimate greater than 10%. Moreover, we statistically adjusted for the influence of some major complications, including such as hyperlipidemia; CHD; AF; DM; and hypertension.

We further analyzed the nonlinearity between the HRR and risk of three-month outcomes using smooth curve fitting. If nonlinearity was detected, we first conducted the saturation effect to find the inflection point. Finally, we performed a subgroup analysis to investigate whether HRR could affect different subgroups, including gender, hyperlipidemia, CHD, AF, DM, and hypertension. In addition, we explored the potential for unmeasured confounding between HRR and risk of three-month outcomes by calculating E-values. Data indicated that the influence of unknown or unmeasured variables on the observed correlation between HRR and risk of three-month outcomes in AIS patients may have been likely minimal. This is because the *E*-value (1.29) is greater than the relative risk of HRR and potential confounding factors (1.14) (13).

## Results

### Population

**Figure 1:**
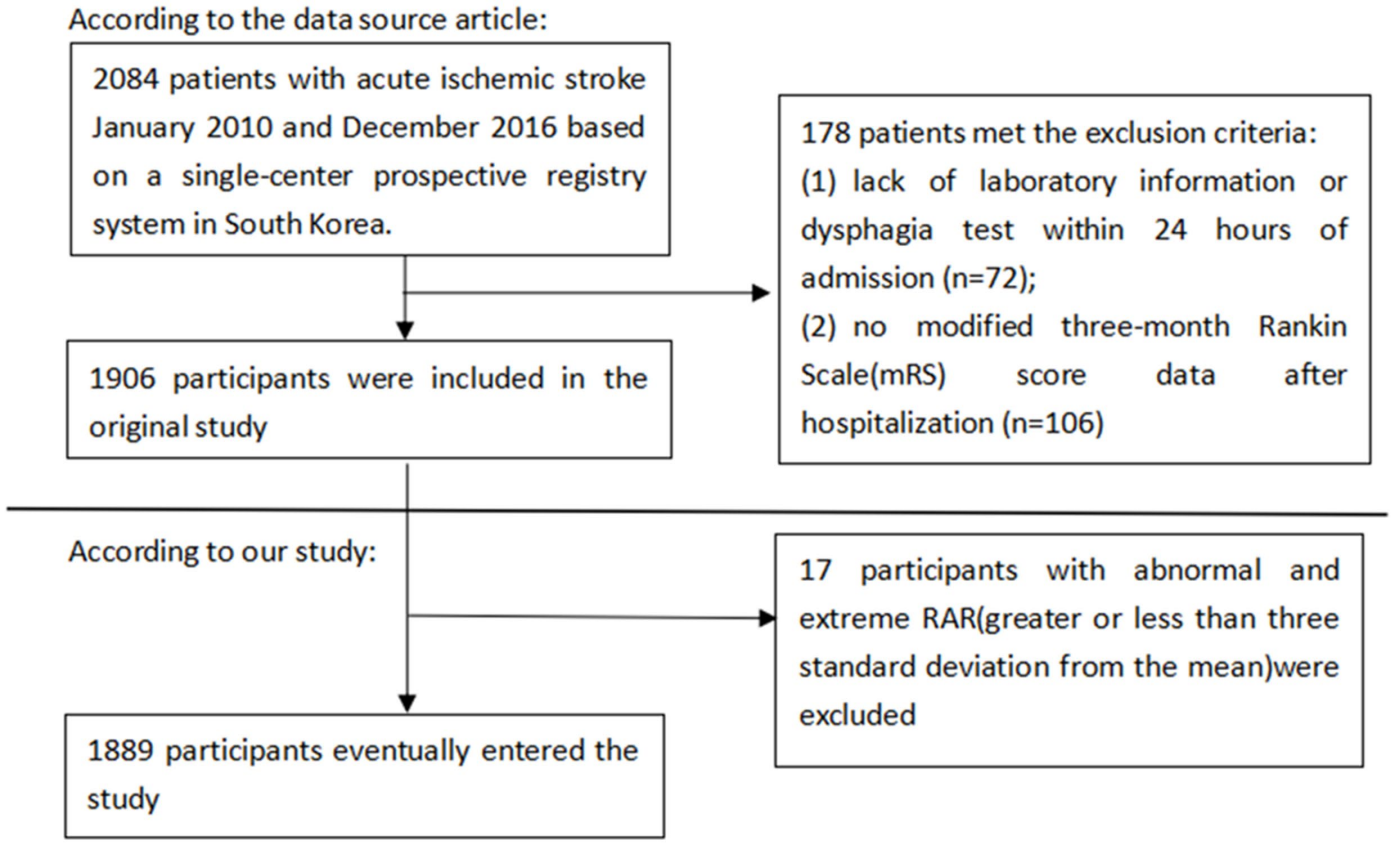
Flow diagram of the patient selection process.

In total, 1,889 patients were enrolled in this study, among whom 1,168(61.28%) were female. The age distribution was as follows: 436(22.88%) patients were under the ages of 60, 505(26.5%) between the ages of 60 and 70, 670(35.15%) patients were between the ages of 70 and 80, and 295(15.48%) patients were over the age of 80. The baseline characteristics grouped according to tertiles of the HRR are presented in Table 1. Patients in the high tertile of HRR had the highest BMI, WBC, PLT, TC, TG, LDL-c, Scr, ALT, and lowest prevalence of comorbidities such as DM, CHD, and AF. Low tertile of HRR value accounted for the highest proportion of low NIHSS and was more likely to smoke (Figure 1).

**Table 1:** Baseline characteristics of participants.

HRR tertiles	All	Low (<9.70)	Middle (9.70–11.21)	High (>11.2)	*p*-value
N		630	628	631	
Age (year)					<0.001
<60	436 (22.88%)	96 (15.24%)	108 (17.20%)	230 (36.45%)	
60–70	505 (26.50%)	122 (19.37%)	200 (31.85%)	179 (28.37%)	
70–80	670 (35.15%)	273 (43.33%)	220 (35.03%)	169 (26.78%)	
≥80	295 (15.48%)	139 (22.06%)	100 (15.92%)	53 (8.40%)	
Sex, (n%)					<0.001
Female	1,168 (61.28%)	278 (44.13%)	368 (58.60%)	513 (81.30%)	
Male	738 (38.72%)	352 (55.87%)	260 (41.40%)	118 (18.70%)	
BMI(kg/m^2^)	23.50 ± 3.25	22.53 ± 3.24	23.48 ± 3.06	24.54 ± 3.15	<0.001
WBC(10^9/L)	8.14 ± 2.89	8.01 ± 3.28	7.93 ± 2.67	8.53 ± 2.63	<0.001
PLT(10^9/L)	223.61 ± 71.31	221.85 ± 87.64	222.61 ± 62.93	227.56 ± 58.88	0.301
TC(mg/dL)	179.26 ± 43.95	168.62 ± 45.44	179.49 ± 40.39	191.02 ± 42.52	<0.001
TG(mg/dL)	105.34 ± 59.97	93.09 ± 50.95	106.59 ± 57.87	116.98 ± 67.31	<0.001
HDL-c(mg/dL)	44.16 ± 16.81	41.61 ± 18.40	47.02 ± 15.64	44.08 ± 15.66	<0.001
LDL-c(mg/dL)	104.15 ± 42.40	95.75 ± 43.76	105.13 ± 39.95	112.75 ± 41.39	<0.001
Scr(mg/dL)	1.09 ± 1.04	1.41 ± 1.68	0.90 ± 0.42	0.95 ± 0.26	<0.001
BUN(mg/dL)	17.60 ± 8.88	20.62 ± 12.11	16.15 ± 5.50	15.80 ± 5.41	<0.001
AST(U/L)	26.11 ± 14.34	26.55 ± 18.25	25.85 ± 13.15	25.68 ± 9.93	0.511
ALT(U/L)	22.38 ± 16.10	19.67 ± 16.37	22.24 ± 14.92	25.20 ± 16.31	<0.001
HGB(g/dL)	13.48 ± 2.00	11.50 ± 1.41	13.72 ± 0.79	15.37 ± 0.98	<0.001
RDW(%)	13.40 ± 1.54	14.27 ± 1.65	13.08 ± 0.66	12.62 ± 0.61	<0.001
Comorbidities, (n%)					
DM	614 (32.21%)	244 (38.73%)	202 (32.17%)	162 (25.67%)	<0.001
CHD	220 (11.54%)	85 (13.49%)	77 (12.26%)	56 (8.87%)	0.029
AF	399 (21.12%)	153 (24.29%)	132 (21.02%)	114 (18.07%)	0.026
Hyperlipidemia	699 (36.67%)	214 (33.97%)	235 (37.42%)	246 (38.99%)	0.168
Hypertension	1,211 (63.54%)	419 (66.51%)	403 (64.17%)	380 (60.22%)	0.064
Smoking	750 (39.35%)	172 (27.30%)	233 (37.10%)	342 (54.20%)	<0.001
NIHSS					<0.001
<5	1,163 (61.02%)	347 (55.08%)	399 (63.54%)	410 (64.98%)	
≥5, <13	493 (25.87%)	174 (27.62%)	158 (25.16%)	156 (24.72%)	
≥13	250 (13.12%)	109 (17.30%)	71 (11.31%)	65 (10.30%)	

BMI, body mass index; WBC, white blood cells; RBC, red blood cells; PLT, platelet; TC, total cholesterol; TG, triglyceride; HDL-c, high-density lipoprotein cholesterol; LDL-c, Low-density lipoproteins cholesterol; Scr, Serum creatinine; BUN, blood urea nitrogen; AST, aspartate aminotransferase; ALT, alanine aminotransferase; HGB, hemoglobin concentration; RDW, red cell distribution width; DM, diabetes mellitus; AF, atrial fibrillation; CHD, coronary heart disease; NIHSS, national institute of health stroke scale.

### Univariate analysis

Our univariate analysis indicated the risk of unfavorable three-month outcomes was decreased in line with rising HRR (Table 2). Specifically, each unit increase in HRR corresponded to a 10% rise in the risk of unfavorable three-month outcomes [OR 0.81, 95% CI 0.81–0.77, *p* < 0.0001]. In addition, age, sex, WBC, Scr, AST, DM, AF, hyperlipidemia, and NIHSS were positively correlated with the risk of unfavorable three-month outcomes. BMI, HDL-c, ALT, hypertension, and smoking were negatively correlated with risk of unfavorable three-month outcomes.

**Table 2:** Univariate analysis.

Variable	Statistics	OR(95% CI), *P*-value
HRR	10.28 ± 1.88	0.81 (0.77, 0.85) <0.0001
Age (year)		
<60	434 (22.98%)	1.0
60–70	501 (26.52%)	1.13 (0.82, 1.56) 0.4402
70–80	662 (35.04%)	1.88 (1.41, 2.52) <0.0001
≥80	292 (15.46%)	3.95 (2.83, 5.50) <0.0001
Sex, (n%)		
Female	1,159 (61.36%)	1.0
Male	730 (38.64%)	1.69 (1.38, 2.07) <0.0001
BMI(kg/m^2^)	23.52 ± 3.26	0.92 (0.89, 0.95) <0.0001
WBC(10^9/L)	8.16 ± 2.89	1.09 (1.05, 1.12) <0.0001
PLT(10^9/L)	224.01 ± 70.97	1.00 (1.00, 1.00) 0.4029
TC(mg/dL)	179.72 ± 43.78	1.00 (0.99, 1.00) 0.0005
TG(mg/dL)	105.56 ± 59.88	1.00 (0.99, 1.00) <0.0001
HDL-c(mg/dL)	44.23 ± 16.76	0.99 (0.99, 1.00) 0.0480
LDL-c(mg/dL)	104.55 ± 42.29	1.00 (0.99, 1.00) 0.0035
Scr(mg/dL)	17.53 ± 8.57	1.02 (1.01, 1.03) 0.0027
BUN(mg/dL)	1.09 ± 1.04	1.02 (0.93, 1.12) 0.7129
AST(U/L)	26.03 ± 14.19	1.01 (1.00, 1.01) 0.0202
ALT(U/L)	22.37 ± 16.03	0.99 (0.99, 1.00) 0.0271
Comorbidities, (n%)		
DM	608 (32.19%)	1.42 (1.15, 1.75) 0.0010
CHD	218 (11.54%)	1.04 (0.76, 1.41) 0.8261
AF	399 (21.12%)	1.97 (1.56, 2.48) <0.0001
Hyperlipidemia	1,202 (63.63%)	1.35 (1.09, 1.67) 0.0055
Hypertension	695 (36.79%)	0.78 (0.63, 0.97) 0.0231
Smoking	747 (39.54%)	0.62 (0.50, 0.76) <0.0001
NIHSS		
<5	1,156 (61.20%)	1.0
≥5, <13	488 (25.83%)	5.67 (4.42, 7.28) <0.0001
≥13	245 (12.97%)	17.36 (12.52, 24.07) <0.0001

BMI, body mass index; WBC, white blood cells; RBC, red blood cells; PLT, platelet; TC, total cholesterol; TG, triglyceride; HDL-c, high-density lipoprotein cholesterol; LDL-c, Low-density lipoproteins cholesterol; Scr, Serum creatinine; BUN, blood urea nitrogen; AST, aspartate aminotransferase; ALT, alanine aminotransferase; HGB, hemoglobin concentration; RDW, red cell distribution width; DM, diabetes mellitus; AF, atrial fibrillation; CHD, coronary heart disease; NIHSS, national institute of health stroke scale.

### Association between HRR and 3-month unfavorable outcomes in patients with AIS

Univariate and multivariate logistic regression models were constructed to evaluate the associations between the HRR and three-month outcomes in patients with AIS. Meanwhile, the non-adjusted and adjusted models are presented in Table 3. When HRR was treated as a continuous variable, the increase in HRR value was associated with a significant decrease in the unfavorable three-month outcomes [OR 0.90, 95% CI 0.84–0.96, P 0.0024]. In addition, the total HRR was converted to a categorical variable (tertile). No changes were observed in the trends, and *p* values in all of the models were < 0.05.

**Table 3:** Univariate and multivariate logistic regression analysis.

Variable	Non-adjusted OR (95% CI), *P*-value	Adjust I OR (95% CI), *P*-value	Adjust II OR (95% CI), *p*-value
HRR	0.81 (0.77, 0.85) <0.0001	0.85 (0.81, 0.91), <0.0001	0.90 (0.84, 0.96), 0.0024
Trisections of HRR			
Low(<9.70)	1.0	1.0	1.0
Middle (9.70–11.21)	0.53 (0.42, 0.68) <0.0001	0.60 (0.47, 0.77) <0.0001	0.67 (0.50, 0.89) 0.0054
High (>11.2)	0.44 (0.34, 0.56) <0.0001	0.60 (0.46, 0.79) 0.0003	0.70 (0.51, 0.96) 0.0258
*P* for trend	0.65 (0.58, 0.74) <0.0001	0.76 (0.66, 0.87) 0.0001	0.83 (0.70, 0.97) 0.0174

Model I: we adjusted for sex and age. Model II: we adjusted for sex, age, BMI, DM, CHD, AF, Hyperlipidemia, Hypertension, and NIHSS.

### Nonlinear relationship between the HRR and 3-month unfavorable outcomes in patients with AIS

**Figure 2:**
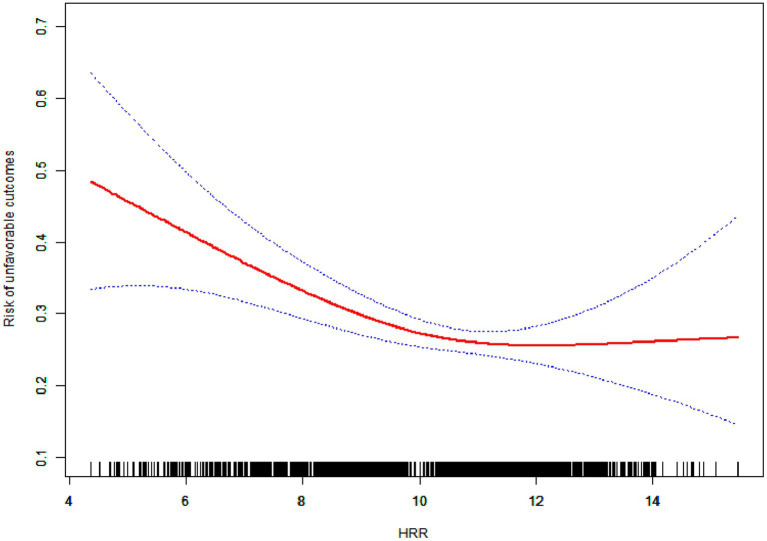
Nonlinearity addressing of HRR and risk of unfavorable outcomes at three-month.

Analysis of the data showed an L-shaped relationship between HRR and the three-month unfavorable outcomes in AIS patients after the smoothing spline fitting was applied and covariates were adjusted for Figure 2. Using a two-segment linear regression model, the inflection point was found at 10.57 (Table 4). When the HRR was≤10.57, the unfavorable three-month outcomes risk decreased by 17% per unit increase in HRR (OR = 0.83, *p* = 0.0001). However, when HRR >10.57, this negative correlation was not detected (OR = 1.10, *p* = 0.2891) (Table 4).

**Table 4:** Two-segment linear regression analysis.

	OR (95%CI), *P*-value
Fitting model by standard linear regression	0.90 (0.84, 0.96) 0.0024
Fitting model by two-piecewise linear regression	
Inflection point of	10.57
≤10.57	0.83 (0.75, 0.91) 0.0001
>10.57	1.10 (0.93, 1.30) 0.2891
*P* for log likelihood ratio test	0.015

We adjusted for sex, age, BMI, DM, CHD, AF, Hyperlipidemia, Hypertension, and NIHSS.

### Subgroup analysis

To validate the stability of our results, subgroup analyses were conducted while considering several confounding variables (Table 5) The interaction test was not statistically significant for sex, age, BMI, DM, CHD, AF, hyperlipidemia, hypertension, and NIHSS (p for interaction = 0.8669, 0.7529, 0.1345, 0.3020, 0.3824, 0.0653, 0.7923, 0.0689, 0.4078, respectively).

**Table 5:** Subgroup analysis of the relationship between HRR and the three-month function outcomes.

Characteristic	OR (95% CI)	*P*-value	P for interaction
Age			0.8669
<60	0.86 (0.76, 0.97)	0.0174	
≥60	0.87 (0.81, 0.93)	<0.0001	
Gender			0.7529
Femal	0.86 (0.79, 0.93)	0.0001	
Male	0.88 (0.79, 0.97)	0.0095	
BMI			0.1345
Low (<23.44)	0.83 (0.76, 0.90)	<0.0001	
High (≥23.44)	0.91 (0.83, 1.00)	0.0442	
HT			0.3020
No	0.83 (0.74, 0.92)	0.0005	
Yes	0.88 (0.82, 0.95)	0.0011	
DM			0.3824
No	0.85 (0.79, 0.92)	<0.0001	
Yes	0.90 (0.81, 0.99)	0.0320	
Hyperlipidemia			0.0653
No	0.83 (0.77, 0.90)	<0.0001	
Yes	0.94 (0.84, 1.06)	0.2461	
CHD			0.7923
No	0.86 (0.81, 0.92)	<0.0001	
Yes	0.89 (0.74, 1.06)	0.1744	
AF			0.0689
No	0.84 (0.78, 0.90)	<0.0001	
Yes	0.95 (0.85, 1.07)	0.4252	
NIHSS			0.4078
<6	0.82 (0.71, 0.95)	0.0066	
≥6	0.88 (0.82, 0.94)	0.0001	

Above model adjusted for sex, age, BMI, DM, CHD, AF, Hyperlipidemia, Hypertension, and NIHSS. In each case, the model is not adjusted for the stratification variable.

## Discussion

Considering the direct association between the central nervous system, blood flow, and tissue oxygen delivery, anemia is postulated to be closely associated with the development and progression of stroke (14). Besides, anemia can up-regulated the levels of inflammatory mediators, thereby lead to poor prognosis of stroke patients (15). In a meta-analysis conducted by Li et al., 13 cohort studies comprising 10,009 stroke patients were included. The study found that anemia increased the risk of death in stroke patients (16). However, Liu et al. found that both low hemoglobin values and high hemoglobin values were associated with adverse stroke outcomes, exhibiting a U-shaped association (17). Some factors influence hemoglobin levels, such as the source of the blood sample, body position, time of day (18), which cause instability when hemoglobin is employed to predict the prognosis of AIS.

RDW is a parameter that reflects heterogeneity of red blood cell size with the potential to influence the prognosis of many diseases, including sepsis, cancer, cardiovascular and cerebrovascular diseases (19–23). After multivariate Cox regression analysis, Xue et al. found that when RDW < 16.7%, it was positively correlated with poor prognosis of stroke (24). He et al. (25) also observed a correlation between higher RDW values measured during different stages of peripheral thrombolysis and an increased risk of hemorrhagic transformation and recurrent strokes. However, the exact mechanisms by which RDW contributes to a poor stroke prognosis remain unclear. Inflammation and oxidative stress are potential underlying factors that warrant further investigation. Inflammatory cytokines can prevent erythropoietin-induced erythrocyte maturation by inhibiting the bone marrow, which is reflected in increased RDW (24). In addition, oxidative stress can disrupt erythropoiesis and alter blood cell membrane deformability and the half-life of RBC in circulation, resulting in high heterogeneity in RBC size (26).

HRR (Hb/RDW) is a new inflammatory marker which stably reflect the degree of oxidative stress and systemic inflammatory response in the body (27). Accumulating evidence has indicated that HRR is a new prognostic indicator of patients with diseases. For example, Liu et al. explored the relationship between patients with non-traumatic subarachnoid hemorrhage HRR values (28). Yılmaz et al. found that HRR is an independent prognostic parameter that predict the progression and survival of patients with metastatic renal cancer (29). Song et al. found that Hb/RDW-SD was negatively linked to the 3-month readmission in elderly heart failure patients (30). In our study, HRR was associated with stroke outcomes at 3 months, with a lower HRR indicating a poorer functional outcome. A piecewise linear regression model was used to calculate the critical inflection point for HRR. We observed a non-linear relationship where, in AIS patients, there was a negative correlation between HRR ≤ 10.57 and the risk of unfavorable outcomes at 3 months, while HRR > 10.57 showed no association with the risk of unfavorable outcomes at 3 months. This non-linear relationship exhibited a saturation effect, indicating that the impact of HRR as an inflammatory factor on AIS prognosis is limited. Therefore, during clinical practice in order to improve patient prognosis we should keep HRR at a high level, but the contribution of increasing HRR to the improvement of AIS prognosis becomes insignificant after HRR exceeds 10.57.

This study has several strengths. Firstly, to the best of our knowledge, this is the first study to explore the association of the HRR with the three-month outcomes in patients with AIS. Secondly, we showed that HRR exhibited a specific non-linear relationship and saturation effect with unfavorable three-month outcomes, and calculated the point. Third, to increase the stability of our results, we conducted subgroup analyses. In addition, we calculated the E-value and confirmed that uncontrolled or unmeasured confounders could not explain our results.

Nevertheless, there are some limitations in this study. First, this is a secondary analysis derived from original data which lacked sufficient information regarding other details of the study population. Therefore, future prospective studies should resolve this problem. Second, since the HRR was only assessed once, it is unclear whether the HRR was altered after some treatment during the hospital stay. This is an important question that need to be further investigated.

The conclusions of this study are expected to guide the application of HRR as a convenient, rapid, and effective tool for predicting three-month outcomes of stroke in AIS patients.

## Data availability statement

The original contributions presented in the study are included in the article/Supplementary material, further inquiries can be directed to the corresponding author.

## Ethics statement

Ethical approval was not required for the studies involving humans because this is a secondary analysis based on another study. The original research was done with the consent of the Institutional Review Board at Seoul National University Hospital.

## Author contributions

XX: Data curation, Methodology, Writing – original draft, Writing – review & editing. KH: Data curation, Methodology, Writing – original draft, Writing – review & editing. YZ: Writing – review & editing. JW: Writing – review & editing.
